# Radiation hybrid maps of the D-genome of *Aegilops tauschii* and their application in sequence assembly of large and complex plant genomes

**DOI:** 10.1186/s12864-015-2030-2

**Published:** 2015-10-16

**Authors:** Ajay Kumar, Raed Seetan, Mohamed Mergoum, Vijay K. Tiwari, Muhammad J. Iqbal, Yi Wang, Omar Al-Azzam, Hana Šimková, Ming-Cheng Luo, Jan Dvorak, Yong Q. Gu, Anne Denton, Andrzej Kilian, Gerard R. Lazo, Shahryar F. Kianian

**Affiliations:** Department of Plant Sciences, North Dakota State University, Fargo, ND 58102 USA; Department of Computer Sciences, North Dakota State University, Fargo, ND 58102 USA; Department of Computer Science, Slippery Rock University, Slippery Rock, PA 16057 USA; Department of Plant Pathology, Kansas State University, Manhattan, KS 66506-5502 USA; Department of Plant Sciences, University of California, Davis, CA 95616 USA; USDA-ARS, Western Regional Research Center, Albany, CA 94710 USA; Department of Computer Science and Information Technology, St. Cloud State University, St. Cloud, MN 56301 USA; Faculty of Science, Palacký University, 783 71 Olomouc, Czech Republic; Institute of Experimental Botany, Šlechtitelů 31, 783-71 Olomouc, Czech Republic; Diversity Arrays Technology Pty Limited, 1 Wilf Crane Crescent, Yarralumla, ACT2600 Australia; USDA-ARS, Cereal Disease Laboratory, University of Minnesota, St. Paul, MN 55108 USA

**Keywords:** *Aegilops tauschii*, Deletion, Physical mapping, Radiation hybrid mapping, Sequence assembly, Wheat

## Abstract

**Background:**

The large and complex genome of bread wheat (*Triticum aestivum* L., ~17 Gb) requires high resolution genome maps with saturated marker scaffolds to anchor and orient BAC contigs/ sequence scaffolds for whole genome assembly. Radiation hybrid (RH) mapping has proven to be an excellent tool for the development of such maps for it offers much higher and more uniform marker resolution across the length of the chromosome compared to genetic mapping and does not require marker polymorphism *per se*, as it is based on presence (retention) *vs*. absence (deletion) marker assay.

**Methods:**

In this study, a 178 line RH panel was genotyped with SSRs and DArT markers to develop the first high resolution RH maps of the entire D-genome of *Ae. tauschii* accession AL8/78. To confirm map order accuracy, the AL8/78-RH maps were compared with:1) a DArT consensus genetic map constructed using more than 100 bi-parental populations, 2) a RH map of the D-genome of reference hexaploid wheat ’Chinese Spring’, and 3) two SNP-based genetic maps, one with anchored D-genome BAC contigs and another with anchored D-genome sequence scaffolds. Using marker sequences, the RH maps were also anchored with a BAC contig based physical map and draft sequence of the D-genome of *Ae. tauschii*.

**Results:**

A total of 609 markers were mapped to 503 unique positions on the seven D-genome chromosomes, with a total map length of 14,706.7 cR. The average distance between any two marker loci was 29.2 cR which corresponds to 2.1 cM or 9.8 Mb. The average mapping resolution across the D-genome was estimated to be 0.34 Mb (Mb/cR) or 0.07 cM (cM/cR). The RH maps showed almost perfect agreement with several published maps with regard to chromosome assignments of markers. The mean rank correlations between the position of markers on AL8/78 maps and the four published maps, ranged from 0.75 to 0.92, suggesting a good agreement in marker order. With 609 mapped markers, a total of 2481 deletions for the whole D-genome were detected with an average deletion size of 42.0 Mb. A total of 520 markers were anchored to 216 *Ae. tauschii* sequence scaffolds, 116 of which were not anchored earlier to the D-genome.

**Conclusion:**

This study reports the development of first high resolution RH maps for the D-genome of *Ae. tauschii* accession AL8/78, which were then used for the anchoring of unassigned sequence scaffolds. This study demonstrates how RH mapping, which offered high and uniform resolution across the length of the chromosome, can facilitate the complete sequence assembly of the large and complex plant genomes.

**Electronic supplementary material:**

The online version of this article (doi:10.1186/s12864-015-2030-2) contains supplementary material, which is available to authorized users.

## Background

Bread wheat (*Triticum aestivum* L.) is a primary staple crop worldwide, as it accounts for a large portion of calories (~20 %) consumed by humans. The complete assembled genome sequence of this important crop holds the key for further guiding improvements in wheat germplasm to meet the needs of an ever increasing world population. However, the ordering of BAC contigs for completing physical maps and assembly of complete genome sequence in large and complex genomes like that of bread wheat, require putting together a highly saturated marker scaffold. For example, the study reporting the first physical map of a wheat chromosome could anchor only 75 % of the chromosome 3B physical map using 1,443 markers [1] suggesting that saturated maps with > 30,000 markers would be needed for complete coverage of the entire wheat genome sequence assembly. High resolution genome maps also serve as effective tools for many other purposes including molecular breeding and positional gene cloning. They provide more choice in selecting quality markers in an important chromosome interval for any marker assisted breeding program and to correctly position a gene of interest between close flanking markers in map-based cloning experiments [2].

In plants, map development thus far has been largely limited to recombination based approaches (genetic mapping). However, recombination mapping techniques generally have three main limitations in developing high density molecular maps. 1) The need for polymorphic markers. Low genetic variation in the cultivated pool reduces the number of markers to be mapped by genetic mapping. This scenario is exacerbated in the case of gene based markers such as ESTs [3], which are valuable for interspecies comparative studies. This is due to the selection pressure which tends to remove any non-advantageous allelic polymorphism in the gene space making them highly monomorphic among individuals of the same species. 2) Uneven distribution of recombination along the length of the chromosomes [4, 5]. Saintenac et al. [4] observed >85-fold difference for crossover frequency per physical distance (cM/Mb) for centromeric bin (C-3BS1-0.33) and sub-telomeric bin (3BS8-0.78-0.87) on wheat chromosome 3B. It was also observed that 42 % of the physical map length of chromosome 3B is represented by only 2.2 % of the genetic map length in the centromeric regions [1]. Consequently, genetic maps lead to inaccurate genetic to physical distance estimates. More importantly, it becomes a difficult (if not impossible) task, to develop high density marker scaffolds for sequence assembly and to clone genes present in low recombination regions when only relying upon genetic mapping. 3) The need for larger population size to achieve higher resolution. The physical and practical considerations of exceptionally large populations can quickly render them unreasonable.

The above challenges are worsened in case of the wheat D-genome, which harbors positive alleles for several important traits [6–10]. Since the D-genome is considered a recent evolutionary addition to the hexaploid wheat genome (>10,000 years ago), there has been limited gene flow from *Ae. tauschii* [11] and, due to this fact, the wheat varieties show limited marker polymorphism among the D-genome loci [3]. Thus, the challenge to develop a saturated D-genome genetic map is intrinsically limited by a dearth of mappable polymorphic markers.

An alternative to recombination-based mapping is radiation hybrid (RH) mapping which uses radiation-induced chromosomal breaks and co-retention pattern of the markers to map them onto chromosomes with the principle that the likelihood of separation of two adjacent markers due to radiation breakage increases with the increase in physical distance [12]. By estimating the frequency of breakage/retention between markers, their relative distance and order can be calculated. Because radiations are expected to cause independent chromosomal breakages, RH mapping offers more uniform resolution across the length of the chromosome in comparison to genetic mapping. It also offers much higher resolution with relatively small number of RH lines [13, 14]. Recently, a RH map constructed using 92 lines showed on average 10X higher resolution than the genetic map (using ~400 doubled haploid lines; [4], and 136X higher resolution in the centromeric regions) [15]. Moreover, RH is based upon presence (retention) *vs*. absence (deletion) assay, allowing the mapping of monomorphic markers as well.

RH mapping, in combination with recombination mapping, has contributed enormously towards whole genome mapping (for review, see Faraut et al. [16]) and sequence assembly of human and animal genomes (for review, see Lewin et al. [17]). However, there have been relatively few examples of RH mapping reported in plants (for review, see Kumar et al. [18]).

Recent advances in next generation sequencing have made it possible to generate huge amount of sequence data in a short amount of time and at a very low cost. Wheat has also benefited from these advances, as evidenced by the recent reports on sequencing and analysis of the whole genome [19], draft sequences of the A-genome progenitor *T. urartu* [20] and the D-genome progenitor *Ae. tauschii* [21]. However, due to the complex nature of the wheat genome, all these studies reported the development of thousands of smaller contigs/scaffolds which ultimately need to be ordered to generate high quality sequence assembly. This clearly shows the need to develop high density genome maps capable of anchoring and ordering all those thousands of contigs/scaffolds. To this end, a RH panel (AL8/78-DGRH_1_) for the D-genome of *Ae. tauschii* accession ‘AL8/78’ [15] was developed to complement ongoing efforts by the members of International Wheat Genome Sequencing Consortium (IWGSC) toward development of a complete physical map (http://avena.pw.usda.gov/RHmapping/, http://www.wheatgenome.org/) of *Ae. tauschii* accession AL8/78 and ultimately bread wheat. Here, an AL8/78-DGRH_1_ panel was used to develop the first high resolution RH maps for the D-genome of *Ae. tauschii* accession AL8/78, which were then used for the anchoring of unassigned sequence scaffolds. This study demonstrates how RH mapping can facilitate the complete sequence assembly of the large and complex plant genomes.

## Results

### Selected RH panel for D-genome of *Ae. tauschii* accession AL8/78

Recently a panel of 1,510 RH lines for mapping of the D-genome of *Ae. tauschii* accession AL8/78 (called AL8/78-DGRH_1_ panel) was developed and characterized [15]. Using 35 SSR markers from across the whole D-genome, a total of 399 RH_1_ lines (called informative lines) were identified, with individual lines showing deletions for at least one of these SSRs. In the present study, 177 informative and one non-informative line (total of 178 lines) were further characterized using the Wheat DArT array (http://www.diversityarrays.com/). DArT analysis resulted in identification of 641 DArT markers specific to the D-genome (present in SW58 and absent in LDN) and showed consistent results on four replicates of each of the parental genotypes, i.e. SW58 and LDN. Analysis of the 178 line DGRH_1_ panel with 641 DArT markers showed an average marker loss of 11.9 %. The average marker loss based on the 35 SSR markers was 7.8 % for these 178 lines. A simple explanation for this difference in deletion frequency could be that SSRs used in this study were mostly associated with QTL/genes and potentially represent gene rich regions. The loss of these regions could lead to reduction in vitality. DArTs span both the genic and non-genic regions of the genome. This could result in reduced detectable deletion frequency for SSRs as compared to DArTs. In our earlier study [15], it was observed that the average marker loss was significantly higher for repeat junction markers (8.9 %), which are present throughout the genome (both in genic and non-genic space), compared to SSRs (3.8 %) and ESTs (3.2 %), which were not significantly different from each other.

Overall, with 676 markers (641 DArTs and 35 SSRs), an average loss of 11.7 % was observed for the whole D-genome. For individual chromosomes, the average marker loss varied from 10.31 % (3D) to 14.46 % (1D) and was found to be homogeneous among the seven D-genome chromosomes (Table 1). However, the average marker loss for individual chromosomes was negatively correlated (r = −0.91) with chromosome size [22].

**Table 1:** Summary of AL8/78 D-genome-RH panel characterization

Chrom.	Average marker loss (%)	Obligate breaks/line^a^	Deletions/line^b^	Max. no of obligate breaks	Average deletion size (Mb)^c^
1D	14.46	3.01 (536)	1.60 (284)	16	42.8 (1–604)
2D	11.76	2.97 (529)	1.56 (278)	15	55.4 (0.9-578.3)
3D	10.31	4.53 (806)	2.43 (432)	20	36.7 (0.8-652.2)
4D	13.57	2.30 (409)	1.428 (227)	10	63.3 (1.3-497.4)
5D	11.56	3.24 (576)	1.71 (304)	17	53.4 (1.0-748.0)
6D	11.54	3.73 (664)	1.99 (355)	17	39.0 (0.9-683.4)
7D	13.00	6.56 (1,167)	3.38 (601)	25	27.3 (0.4-692.5)
D-genome	11.70	26.33 (4,687)	13.94 (2,481)	97	42.0 (0.4-748)

^a^Number in parenthesis is the total number of obligate breaks in a panel of 178 RH lines

^b^Number in parenthesis is the total number of deletions in a panel of 178 RH lines

^c^Number in parenthesis is the range of deletion size

The deletion frequency for individual markers varied from 0.6 to 26.7 % and was heterogeneous across the whole genome. However, *χ*^2^ homogeneity tests showed that the deletion frequency of individual markers in a given chromosome was homogeneous (*p* ≤ 0.001) for chromosomes 1D, 2D, 4D, 5D and 6D, while it was heterogeneous for chromosomes 3D and 7D. In case of chromosome 3D, heterogeneity was mainly due to few markers present together on the long arm and less than expected deletion frequency, while in case of chromosome 7D, the heterogeneity was mainly caused by few markers present on the short arm and higher than expected deletion frequency.

The average marker loss for individual DGRH_1_ lines, based on 676 markers, ranged from 0 to 90 %. A high correlation (r = 0.64, *p* < 0.000001) was observed between the deletion frequencies of the RH lines based on characterization with 35 (SSRs) and 641markers (DArTs).

**Fig. 1:**
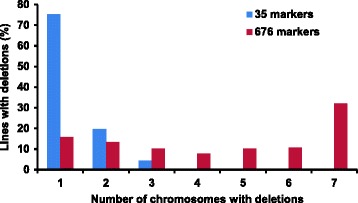
Characterization of the RH panel with two different set of markers

In the 178 DGRH_1_ line panel, 35 SSRs detected deletions for only one chromosome in about three fourth (75.3 %) of the lines, while the remaining lines showed deletions for two or three chromosomes only. However, characterization of the same panel with 676 markers (641 DArTs and 35 SSRs) showed that majority of these lines have deletions for multiple chromosomes (Fig. 1). About one third (32 %) of the lines showed deletions for all seven D-genome chromosomes and >84 % lines showed deletions for two or more chromosomes.

### RH maps for D-genome of *Aegilops tauschii*

**Fig. 2:**
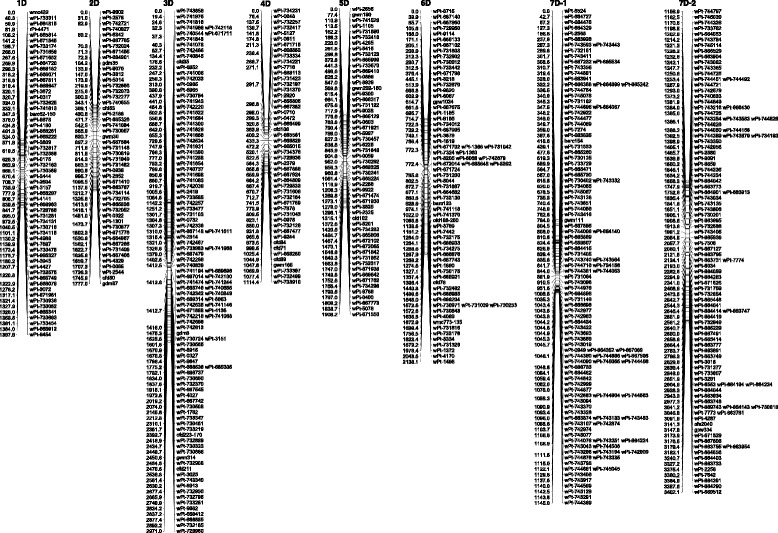
Radiation hybrid maps of D-genome of *Aegilops tauschii* accession AL8/78. The chromosome 7D has also one contiguous RH map but due to larger size, it has been split into two parts (7D1 and 7D2)

The 641 DArT and 35 SSR marker data [15] was used to generate RH maps for the D-genome. In total, 609 markers (580 DArTs and 29 SSRs) could be mapped to 503 unique positions on the seven chromosomes (Table 2; Fig. 2). The number of markers mapped on individual chromosomes ranged from 49 (4D) to as many as 218 (7D). The 106 co-segregating markers belong to six chromosomes (all except 5D); chromosome 7D had the highest number (52) and chromosome 5D had no such markers.

**Table 2:** Statistics on RH maps of all seven D-genome chromosomes of *Aegilops tauschii* accession AL8/78

Chromosome	Markers Mapped	Unique Loci	Total Map Length (cR)	Marker Density (cR/marker)	Map Resolution	
					cM/cR^a^	Mb/cR^b^
1D	58	55	1,397.9	25.4	0.114	0.432
2D	49	46	1,777.0	38.6	0.065	0.409
3D	108	81	2,971.0	36.7	0.058	0.259
4D	49	43	1,114.4	25.9	0.104	0.581
5D	55	55	1,908.2	34.7	0.085	0.392
6D	72	57	2,136.1	37.5	0.063	0.333
7D	218	166	3,402.1	20.5	0.052	0.214
Whole D-genome	609	503	14,706.70	29.2	0.070	0.34

^a^Genetic distances based on Huang et al. [23]

^b^Physical sizes based on Doležel et al. [22]

The 609 markers (or 503 loci) covered a total map length of 14,706.7 cR (1,034.5 cM, based on consensus DArT genetic map by Huang et al. [23]) with individual chromosome lengths ranging from 1,114.4 cR (chromosome 4D) to 3,402.1 cR (chromosome 7D) (Table 2). The average distance between any two marker loci was 29.2 cR, which corresponds to 2.1 cM [23] or 9.8 Mb [22]. The highest marker density was observed for chromosome 7D, which has an average distance of 20.5 cR between any two marker loci, while the lowest markers density was observed for chromosome 2D, with an average distance of 38.6 cR between any two marker loci.

#### Resolution of the AL8/78-RH maps

One of the most important features in any map is the mapping resolution. In RH mapping, the resolution may be expressed either as Kilobases (Kb) to centiRay (cR) ratio or centimorgan (cM) to centiRay (cR) ratio. So, the RH map distances were compared with the estimated physical sizes of wheat D-genome chromosomes [22] and genetic distances [23] on the D-genome map (Table 2). The estimate for Mb/cR was 0.34 for the whole D-genome, with a range of 0.21 (7D) to 0.58 (4D) for individual chromosomes. Similarly, the estimate for cM/cR were 0.07 for the whole D-genome, with a range of 0.052 (7D) to 0.114 (1D). A high correlation (r = 0.75, *p* < 0.05) was observed between the resolution (Mb/cR) and the number of marker loci mapped on individual chromosomes, meaning that the resolution was higher for chromosomes with more mapped loci. This is expected for the resolution in RH mapping depends upon the number of deletions, and the present study as well as the past studies [15, 24, 25] clearly show that with few markers only a few deletions are detected in the RH lines. The increase in marker density leads to the detection of more deletions, thus resulting in higher resolution.

#### Anchoring the AL8/78-RH map with BAC contig assembly and draft genome sequence

In order to achieve a completely assembled genome sequence for the wheat D-genome, two important studies were recently published. Luo et al. [5] generated a genetic map with 7,185 markers (referred to as AL8/78 × AS75 genetic map hereafter) and then anchored a total of 4.03 Gb of D-genome BAC contigs to the map, while Jia et al. [21] sequenced *Ae. tauschii* genome to ~90X depth of short reads, and assembled them into sequence scaffolds which represented 83.4 % of the D-genome. In order to demonstrate the utility of RH maps in anchoring BAC contigs or sequence scaffolds, the present RH maps were used as a framework to anchor a subset of the *Ae. tauschii* D-genome physical maps and draft genome sequence by BLASTN comparison of the available sequences of 531 DArT markers against the marker sequences mapped in AL8/78 × AS75 genetic map [5] and draft genome sequence of *Ae. tauschii* [21].

A total of 31 marker sequences showed matches (score > 500) with the markers mapped in the AL8/78 × AS75 genetic map of Luo et al. [5] (see Additional file 1 for details). Seven contigs each were anchored to chromosomes 3D and 4D, four contigs each were anchored to chromosomes 2D, 5D and 6D, and three contigs were anchored to chromosome 1D, while only two contigs were anchored to chromosome 7D (Table 3). Of these 31 markers, 27 markers were mapped to the same chromosome in our RH maps and genetic maps of Luo et al. [5], while four markers were mapped to different chromosomes in the two maps.

**Table 3:** Summary of the AL8/78-RH map markers anchored to D-genome sequence scaffolds and BAC contigs

	Anchored to sequence scaffolds^a^		Anchored to BAC contigs^b^	
Chromosome	No. of DArT markers	No of scaffolds (Unique)	No. of DArT markers	No. of BAC contigs
1D	46	24	3	3
2D	41	25	4	4
3D	93	37	7	7
4D	39	25	7	7
5D	48	30	4	4
6D	58	35	4	4
7D	195	40	2	2
Whole D-genome	520	216	31	31

^a^Jia et al. [21]; ^b^Luo et al. [5]

A total of 520 (out of 531) DArT sequences showed positive hits with the draft genome sequence of *Ae. tauschii* (see Additional file 1 for details). These 520 markers were anchored to 216 sequence scaffolds (Table 3, see Additional file 1 for details). Among them, 126 scaffolds were singularly anchored to DArT marker, while the remaining 90 scaffolds were anchored to multiple (2–52) markers. The number of sequence scaffolds anchored to individual chromosomes ranged from 24 (1D) to 40 (7D). Among the 216 sequence scaffolds which were anchored to AL8/78-RH map, only 100 scaffolds (anchored to 234 markers) were previously anchored and ordered by Jia et al. [5] using a population of 490 F_2_ individuals derived from a cross between the *Ae. tauschii* accessions Y2280 and AL8/78. We were able to anchor and order an additional 116 sequence scaffolds (anchored to 286 markers) using RH maps. In general, a good agreement in marker order was observed between AL8/78-RH maps and AL8/78 × AS75 or Y2280 × AL8/78 genetic maps.

#### Comparison of AL8/78-RH maps with other published genetic and RH maps

The accuracy of the marker order can be tested by comparing common markers mapped in different maps. So, the marker order of AL8/78-RH maps was tested by comparison with: 1) a DArT consensus genetic map [23]; 2) a SNP based genetic map with anchored D-genome BAC contigs [5]; 3) a SNP genetic map anchoring sequence scaffolds of D-genome (referred as Y2280 × AL8/78 map hereafter [21]); and 4) a RH map of D-genome of Chinese Spring (referred as CS RH map hereafter [14]).

The AL8/78-RH maps were directly compared with the consensus maps by Huang et al. [23] and the RH maps by Tiwari et al. [14] as all these maps have DArT markers with the same nomenclature. In order to identify the common markers mapped on the *Ae. tauschii* genetic map by Luo et al. [5], the available 523 DArT sequences mapped in AL8/78-RH map were used to BLAST compare against the sequences containing SNP markers positioned on the genetic map [5]. In the case of Jia et al. [21], the SNP markers were used to anchor the sequence scaffolds onto the Y2280 × AL8/78 genetic map. So, to identify the common markers, a BLAST query was performed with the DArT sequences (mapped in AL8/78-RH map) against the mapped sequence scaffolds.

In relation to the AL8/78-RH maps, there are 114 markers in common with the DArT consensus genetic map [23], 285 markers in common with the CS-RH map [14], 31 markers in common with the BAC contig-anchored *Ae. tauschii* genetic map (Luo et al. 2013) and 236 markers in common with sequence scaffold-anchored *Ae. tauschii* map [21]. In total, 75 % of DArT markers (435 out of 580) mapped in the present study were present on at least one of the above four maps. The remaining 155 DArT markers mapped in the AL8/78-RH map were not present in any of the four previous studies mentioned.

Overall, a good consistency in chromosome assignment and marker order was observed in the comparative maps. All the markers in common between the AL8/78-RH maps and the consensus genetic [23] and CS-RH maps [14] showed complete agreement in chromosome assignment of the markers. Among the 31 markers common between the AL8/78-RH maps and the AL8/78 × AS75 genetic maps [5], only four markers {wPt-730660 (3D), wPt-9820 (6D), wPt-665921 (6D), wPt-671626 (7D)} were assigned to different chromosomes in the two maps. However, these four markers showing different chromosome assignment in the AL8/78 × AS75 genetic map, when checked in other published maps [14, 21, 23], were found to be mapped on the same chromosomes as that of the present RH maps. The markers wPt-730660 and wPt-665921 were mapped on 3D and 6D respectively by Tiwari et al. [14], the marker wPt-9820 was mapped on 6D by both Tiwari et al. [14] and Jia et al. [21], on the same chromosomes as the present RH maps. The marker wPt-671626 (7D) was not mapped in any other map but did show positive hybridization to chromosome 7D, when hybridized with genomic representations of flow-sorted chromosome arms (cv. Chinese Spring) of bread wheat (unpublished data), consistent with the AL8/78-RH map data.

Among the 236 markers in common between AL8/78-RH maps and Y2280 × AL8/78 genetic maps, only eight markers {wPt-4988 (1D), wPt-7697 (1D), wPt-9662 (3D), wPt-669412 (3D), wPt-731423 (4D), wPt-732197 (4D), wPt-731370 (4D), wPt-2325 (5D)} showed different chromosome assignments between the two maps. However, further investigations showed that three {wPt-9662 (3D), wPt-669412 (3D), wPt-2325 (5D)} out of the eight markers were mapped by Tiwari et al. [14] and/or Huang et al. [23] on the same chromosomes as that of the AL8/78-RH maps. The remaining five markers were not mapped in any other maps. However, three of the remaining five markers {wPt-4988 (1D), wPt-7697(1D), wPt-731423 (4D)} showed positive hybridization to the same chromosome on which they were mapped in our RH maps, when hybridized with the flow-sorted chromosome arms (unpublished data). The marker order in AL8/78-RH maps also showed a good agreement with all the above mentioned maps. Majority of the markers with disparity in order showed minor local flipped rearrangements. There were only few markers which showed large differences in placement on the chromosomes in comparison with some of the earlier studies. An agreement in map position can be estimated by calculating the rank order correlations between the map position of AL8/78-RH maps and other published maps. The mean rank order correlations between the map position of each chromosome of AL8/78-RH maps with the Y2280 × AL8/78 genetic map [21], AL8/78 × AS75 genetic map [5], DArT consensus genetic map [23] and CS-RH map [14] averaged 0.92 (range of 0.85-0.96), 0.89 (range of 0.65-1.0), 0.86 (range of 0.65-1.0) and 0.75 (range of 0.36-0.97), respectively (Table 4). Further analysis on the data suggests that the few low values of rank order correlations between map positions of chromosomes are mainly due to a small number of common markers as well as due to several co-segregating markers present in maps other than the AL8/78-RH maps. However, overall these comparisons with multiple studies clearly validate the accuracy of AL8/78-RH maps.

**Table 4:** Rank correlation coefficient values between the map position of each chromosome of AL8/78-RH maps with four other published maps of D-genome

Chromosome	Y2280 × AL8/78 genetic map^a*^	AL8/78 × AS75 genetic map^b*^	DArT consensus genetic map^c*^	CS-RH map^d*^
1D	0.85 (0.0004)	1.0 (0.15)	1.0 (0.01)	0.84 (0.00)
2D	0.98 (0.003)	1.0 (0.31)	0.65 (0.008)	0.90 (0.07)
3D	0.96 (0.0)	0.77 (0.08)	0.96 (0.0)	0.97 (0.0)
4D	0.94 (0.0002)	0.89 (0.02)	-	0.80 (0.14)
5D	0.96 (0.0)	0.65 (0.25)	0.81 (0.06)	0.76 (0.004)
6D	0.89 (0.0)	1.0 (0.31)	0.88 (0.03)	0.36 (0.07)
7D	0.86 (0.0)	-	0.87 (0.0)	0.59 (0.0)
Average	0.92	0.89	0.86	0.75

^a^Jia et al. [21]; ^b^Luo et al. [5]; ^c^Huang et al. [22]; ^d^Tiwari et al. [14]

*Figures in parenthesis are the probability values; higher *p-*values in all cases were due to fewer common markers between the compared maps

### Size of deletions in the AL8/78-RH panel

To estimate the number of deletions (or obligate breaks), all the data belonging to mapped markers was arranged in map order for each line. An obligate break was scored between two loci (a and a + 1) whenever the score for marker loci a =1 and for a + 1 = 0 or vice versa. With 609 mapped markers, a total of 4,687 obligate breaks including 275 terminal and 4,412 interstitial breaks were observed across the whole D-genome. The average number of obligate breaks per line was 26.33 (Table 1), with a maximum of 97 breaks in a single line for the whole D-genome. For individual chromosomes, the average number of obligate breaks per line ranged from 2.30 (4D) to 6.56 (7D). The number of obligate breaks per chromosome were homogeneous among chromosomes (*P* = 0.001).

One obligate break is required for a terminal deletion, while two obligate breaks are required for an interstitial deletion. Based on 609 mapped markers, 4,672 breaks yielded a total of 2,481 deletions, including 275 terminal and 2,206 interstitial deletions for the whole D-genome. The average number of deletions in the whole D-genome for a single line was 13.87 with a maximum of 51 deletions for a single line. The average number of deletions per line for individual chromosomes was homogeneous (*P* = 0.001) and ranged from 1.45 (4D) to 3.16 (7D).

The number of obligate breaks/deletions, among other factors, depends on chromosome length and number of markers used for characterization. In the present study, both the number of markers and the physical length of the chromosomes had a significant correlation with the average number of deletions detected for a particular chromosome. However, the correlation between the number of markers and number of deletions (r^2^ = 0.97, *P* < 0.001) was much higher as compared to the correlations between physical length of the chromosome and number of deletions (r^2^ = 0.46, *P* = 0.14).

To estimate the size of deletions, the physical size of the deletions for individual chromosomes was estimated first in RH map distances (cR) and then converted into physical distances (Mb) using the physical sizes of individual chromosomes [22]. Individually, the size of deletions ranged from 0.4 to 748 Mb (Table 1). However, the average deletion size for the whole D-genome was 42.0 Mb, while for individual chromosomes it varied from 27.3 Mb for chromosome 7D to 63.3 Mb for chromosome 4D (Table 1). A very high negative rank order correlation (−0.97, *P* < 0.05) was observed between the number of markers and the average deletion size for a particular chromosome. This is expected because with few markers mostly larger deletions are detected. However, an increase in marker density leads to detection of smaller deletions which ultimately reduces the average size of deletions.

**Fig. 3:**
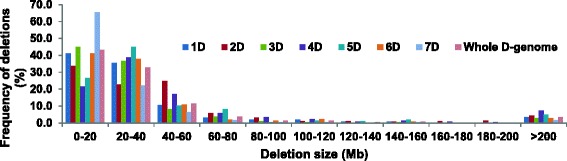
Distribution of deletion sizes in AL8/78-DGRH_1_ panel

Based on 609 marker data, the frequency of deletions of different sizes in the RH panel was almost similar for different chromosomes (Fig. 3). Deletions of <20 Mb form the major group followed by deletions ranging from 20 to 40 Mb. Together, the deletions of sizes <40 Mb represent about 77 % of the total deletions detected for the whole D-genome. A small proportion (3.6 %) of deletions of sizes larger than 200 Mb was also observed.

## Discussion

Sequencing costs have decreased radically in the past few years. However, it still is not possible to completely assemble short sequence reads of large and complex plant genomes such as wheat. So, to achieve the goal of an accurate sequence assembly of wheat, several approaches are being followed. Recently, several studies aimed at achieving the complete genome sequence of the D-genome of wheat were published [5, 21]. Luo et al. [5] fingerprinted BAC clones which were then assembled into contigs, while Jia et al. [21] used a very high depth of short sequence reads to develop sequence contigs/scaffolds. However, in both cases, a high density genome map is needed to anchor, order or align the large number of contigs/scaffolds onto the chromosomes. Therefore, the availability of a high quality genome map is of utmost importance (at least with the existing technologies) in generating a correct genome assembly in species with large and complex genomes. In this study, we characterized a RH panel in depth and demonstrated how development of a RH map can contribute to the assembly of large and complex genomes.

### Characterization of radiation induced deletions

The majority of the informative lines when initially selected with few markers (35) generally showed deletions for a single chromosome [15], but when characterized with more markers (641) in the present study, deletions for multiple chromosomes were detected (Fig. 1). The reason for this discovery of additional affected chromosomes could be that the majority of the deletions caused by gamma rays are small in size (Fig. 3) and are not detected when using only a few widely spaced markers. However, when more markers are used the deletion detection efficiency across the genome is improved. This also is supported by a high correlation (r = 0.97, *P* < 0.001) observed between the number of detected deletions on any chromosome and density of markers mapped on that particular chromosome. Similar results were previously reported [15, 24, 25]. It indicates that informative lines might have multiple deletions for all chromosomes requiring high density of markers for detection.

Based on 609 mapped markers, the average deletion size was estimated to be 42 MB at the whole genome level; however, deletion size differed widely amongst the seven D-genome chromosomes. These differences could be attributed mainly to the different number of markers mapped on each chromosome (Table 2). The increase in number of markers used for genotyping leads to the detection of additional smaller deletions, which ultimately results in decreased average deletion size determination. This observation is evident in a very high rank order correlation (−0.97) between the number of markers and the average deletion size for a particular chromosome. This also means that the estimates of average deletion size are likely to decrease further when these RH lines are genotyped with more markers.

When the deletions were grouped based on size, the frequency of deletions of different sizes showed almost similar patterns for all chromosomes (Fig. 3). The majority of deletions on all chromosomes fall into two smallest size groups, 0–20 and 20–40 Mb. However, it was interesting to observe that in the 0–20 Mb group, the proportion of deletions was positively correlated with the number of marker loci mapped on a particular chromosome. But, as we move towards larger size deletion groups (21–40, 41–60, 61–80 Mb), a highly significant negative correlation between proportion of deletions and number of marker loci mapped on a particular chromosome was observed. These observations indicate that an increase in number of markers for genotyping will lead to the detection of additional smaller deletions, which correspondingly means a decrease in the proportion of larger deletions. These smaller deletions will be the key in developing high resolution genome maps for whole genome sequence assembly.

### RH maps of *Ae. tauschii* genome and mapping resolution

The accession AL8/78 was chosen for it has been used extensively for genomic studies including the sequencing of D-genome chromosomes [5, 21]. The RH maps reported here demonstrate the utility of RH methodology in achieving higher resolution across the length of the chromosome, anchoring and ordering BAC contigs/sequence scaffold and identification of potential errors in genetic maps. One of the factors affecting the RH map resolution is the panel size. The AL8/78-RH maps were developed using a total of 178 RH lines. Most of the studies in animals, however, have used about 100 RH lines (for review, see Faraut et al. [16]). There were two main reasons to use a larger RH panel when developing high density RH maps in plants. First, compared to the animal RH panels, most of the plant RH panels have lower deletion frequency as most are represented by viable plants. The average marker deletion frequency of the RH panel used in this study was 11.7 %, while most of the animal panels have a reported 70-80 % deletion frequency (for review, see Faraut et al. [16]). Secondly, studies have shown that utilizing panels with larger number of lines yield fewer linkage groups and provide more accurate information [26, 27]. Thus, we believe that the use of comparably larger RH panels in plants might be one of the possible solutions towards achieving the resolutions and level of success for complete genome mapping.

In this study, a total of 609 markers (580 DArTs and 29 SSRs) were mapped to 503 unique loci (82.6 %). Comparatively, a study reporting the consensus maps of the D-genome of wheat based on the genetic data from about 100 populations could resolve only 42.2 % of the DArT loci [23]. This shows the power of RH lines in resolving the closely linked markers. In the present study, the main reason for the co-segregation of ~17.4 % of the markers with other loci was some level of redundancy in genomic representation of DArT markers. When the available sequences of 530 DArT markers were analyzed for redundancy, it was observed that 14.7 % of the markers were redundant (37 sequences were represented by 115 DArT markers). A recent study which analyzed sequences of DArT markers from the A and B genomes of wheat, also estimated that about 13.6 % of the markers showed sequence similarity with other markers [28]. This suggests that the present set of RH lines was able to resolve majority (>97 %) of the unique loci.

The precision of an RH map can be characterized by the resolution, expressed in the kilobase (kb) to centiRay (cR) ratio. The present maps display an average mapping resolution of 0.34 Mb/cR. In comparison to genetic maps, it was 0.07 cM/cR. Therefore, our RH map resolution is higher than the resolution reported for chromosome 3B (0.53 Mb/cR; [13], which could be attributed to the larger RH panel used in the present study. Highest resolution was observed for chromosome 7D which has the largest number of mapped markers. This is likely due to interdependence of the number of breakages/deletions and the number of markers used to detect them. This is also evident from a high positive correlation between the number of obligate breaks (r = 0.88) or resolution (r = 0.75) and the number of markers loci mapped on individual chromosome.

### Accuracy of AL8/78-RH maps

The accuracy of the AL8/78-RH maps was tested for both chromosome assignment and marker order by comparing with previously published genetic and RH maps [5, 14, 21, 23]. The AL8/78 RH maps were in complete agreement in chromosome assignment with the consensus genetic maps by Huang et al. [23] and CS-RH maps by Tiwari et al. [14]. This observation is expected as each of these studies used the DArT platform for genotyping. Only a few markers (<5 %) showed different chromosome assignments from the genetic maps by Luo et al. [5] and Jia et al. [21]. A simple reason for these differences in chromosomal assignment could be that the three studies used different genotyping methods and as wheat has a complex and highly repetitive genome, depending on genotyping method different orthologous or paralogous sequences could be amplified resulting in different assignment of marker. However, the results of AL8/78 RH mapping were confirmed when these few discrepancies were further investigated by comparison with other studies (other than the one which showed discrepancies) and/or hybridization with the genomic representations of flow-sorted chromosome arms of bread wheat. The results showed that most of these questionable markers (10 out of 12; for remaining two markers no additional information could be obtained) respect the chromosome assignment of AL8/78-RH maps. This shows the potential of RH mapping approach for correcting genotyping errors.

In terms of marker order, there was a good agreement between the AL8/78-RH map and other available maps. The few differences in marker order and local inversions observed could be due to several reasons including different methods of genotyping, differences in genetic material, presence of repeat elements/paralogues, different analysis algorithms and parameters used in different studies for mapping. More specifically, the differences observed with DArT consensus genetic map could be attributed to the fact that the consensus map is based on the data from about 100 bi-parental populations, which may have different structural and recombination frequency variations. Similarly, the differences with CS-RH map could be attributed to differences on D-genome as a result of 10,000 years of evolution.

The AL8/78-RH maps showed highest values of rank order correlation when compared with Y2280 × AL8/78 and AL8/78 × AS75 genetic maps, which could be expected as both of these genetic maps used a mapping population developed using AL8/78 as one of the parent [5, 21]. The few differences observed in marker order could be attributed to different genotyping methods used in these studies. However, as all the mapping approaches are prone to minor errors, information from several sources including genetic and radiation hybrid maps may help in achieving high quality genome sequence assembly.

### RH mapping increases the number of mappable markers

One of the major constraints in developing high density maps using recombination mapping is the dependence upon polymorphism. In wheat, most of the mapping populations show about 20-40 % genetic polymorphism [29, 30], which further decreases when mapping gene based markers (9.9 % for EST-derived SSRs *vs* 35.5 % for genomic SSRs [3]). The above challenge becomes even worse in the case for the wheat D-genome, since it is the most recent evolutionary addition to the hexaploid wheat, and there has been limited gene flow from *Ae. tauschii* [11]. Due to this fact, the current wheat germplasm show much lower molecular marker polymorphism for the D-genome loci, compared to the A or B genomes [3]. Thus, any attempts to develop saturated maps for the wheat D-genome using genetic mapping confront a persistent problem of having limited mappable polymorphic markers. On the other hand, RH mapping does not rely on allelic polymorphism and uses assays for the presence and absence of marker loci, thus making it possible to map monomorphic loci.

The published genetic mapping studies have reported very few polymorphic markers for the D-genome [31, 32]. Using bi-parental genetic populations, a total of 41 DArT markers by Akbari et al. [31], and 55 markers by Wang et al. [32] were mapped on D-genome of wheat using DArT array. The study by Sorrells et al. [33] was able to map 219 DArT markers on the D-genome of the ITMI population as this population was developed using a synthetic wheat with the D-genome of *Ae. tauschii*. A recent study which integrated the mapping data from more than 100 bi-parental genetic populations to develop a high-density DArT consensus map had a total of only 851 DArT markers placed on D-genome [23]. Compared to the above mentioned genetic mapping studies, using only a 178 RH line population, we were able to map 580 DArT markers, while another RH study was able to map 671 DArT markers using a panel for the D-genome of Chinese Spring [14]. In total, both these studies were able to map 966 DArT markers (285 being in common to both studies) onto the D-genome of wheat. This clearly shows that with RH mapping populations, the number of mappable markers can be increased several fold over conventional genetic mapping methods. This advantage will be important toward obtaining a complete marker scaffold for developing a whole genome assembly in wheat and other complex plant genomes.

### Radiation hybrids offer more uniform mapping

**Fig. 4:**
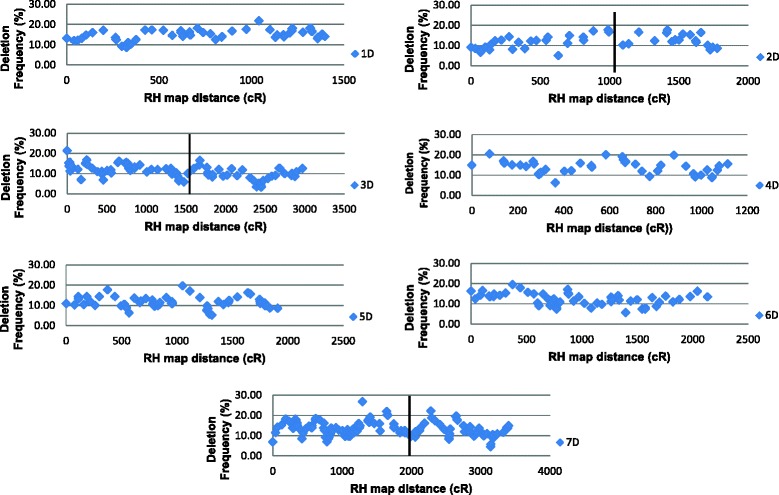
A scattergram of the marker retention frequencies along D-genome chromosomes. The horizontal line represents the approximate position of the centromeres as estimated by hybridization of DArT sequences with the genomic representations of flow-sorted chromosome arms of bread wheat

Another critical issue with genetic mapping is the non-uniform distribution of recombination along the length of a chromosome. It is also well established that a large portion of the chromosomes around the centromere in plants generally show very low recombination or no recombination [4, 34] and this holds true with the D-genome of wheat [5]. Luo et al. [5] observed that although the genes were distributed across the entire length of *Ae. tauschii* chromosomes, the average recombination rate ranged from almost zero in proximal chromosome regions to about 1.5-2.0 cM/Mb in the distal regions. Therefore, using genetic mapping, it becomes very difficult, if not impossible, to develop high resolution maps for anchoring sequence scaffold/contigs and to clone genes/QTL from the low recombination regions, particularly in the regions near the centromere. Comparatively, RH maps show more uniform and complete coverage of the genome as they use radiation induced deletions rather than genetic recombinations [13, 15]. The results of this study also showed that the deletion frequency for individual markers was homogeneous for majority of the chromosomes and no significant differences in deletion frequency were observed across a given chromosome (Fig. 4). The genetic maps of the D-genome of AL8/78 showed that almost no recombination occurred in the proximal regions of all chromosomes [5]. However, the physical maps of AL8/78 [5] show that the genes are distributed across the length of the chromosomes and the regions of high gene density are interspersed by regions of low gene density. A somewhat similar pattern was observed for deletion frequency of markers for all the chromosomes, where regions of high deletion frequency were interspersed by regions of low deletion frequency (Fig. 4). The reason could be that the most of the DArT markers represent gene sequences [28] and the irradiated cells with deletions in gene rich regions are less likely to survive the selection during RH plant development. This could mean that the observed deletion frequency for gene rich regions will be less, while it will be higher for low gene density regions.

Additional evidence of a more uniform distribution of markers in RH maps was observed when AL8/78-RH maps were compared with genetic maps [5, 21, 23]. The analysis shows several blocks of low marker coverage on most of the chromosomes in genetic maps in comparison to our RH maps. This observation was clearly visible on chromosomes 3D and 7D which have dense RH maps (Additional file 1). However, when AL8/78-RH maps are compared with CS-RH maps [14], most of the chromosomes show much better uniform distribution of markers in both the RH maps (Additional file 1). Another evidence for a more uniform distribution of markers in RH maps was the number of marker loci mapped on individual chromosomes. Among the D-genome chromosomes, most of the genetic mapping studies were able to map only few markers on chromosomes 4D and 5D compared to other chromosomes due to lack of polymorphism for these chromosomes [23, 31, 33]. Comparatively, in the present study, if the chromosomes 3D and 7D which have more markers because the DArT array was enriched with these chromosome specific genomic representations (unpublished data) are excluded, the remaining five chromosomes of the AL8/78-RH maps have almost similar number of mapped loci. This clearly shows that RH maps have great potential to complement genetic mapping approach for developing high density genome maps for the purpose of assembly of large plant genomes as well as for cloning genes in low recombination regions.

### AL8/78-RH maps for sequence assembly

In last few years with the rapid developments in DNA sequencing technologies, more than fifty plant genomes have been published [35]. However, still it is not possible to *de novo* generate one contiguous sequence for a given chromosome using only next generation sequencing data in large and complex genomes. In wheat, recently published draft sequence assemblies of the A- [20] and D-genome [21] reported the generation of thousands of scaffolds with a N50 length of only 63.69 kb and 57.6 kb, respectively. Another recent study using BAC fingerprinting, generated 3,153 BAC contigs [5]. In both cases, a high resolution genome map is needed to anchor and order these scaffold and contigs for high quality genome assembly. Given the high and uniform resolution of AL8/78-RH maps and the fact that a larger panel comprised of 400 informative lines (only 177 were used in the present study) is now available through this project [15], the use of RH might be promising approach to anchor and order most of the sequence scaffolds of the 4.36-Gb D-genome.

## Conclusion

A radiation hybrid panel was used to construct maps for the genome of *Ae. tauschii* accession AL8/78. The RH maps showed good accuracy when compared with several other independent genetic and RH data sets. These RH maps showed a mapping resolution of 0.34 Mb and 0.07 cM per cR. The RH approach was able to map almost three times more markers compared to the available genetic maps of the D-genome and provided much more uniform distribution of markers across the genome. The panel of only 178 RH lines was able to resolve >97 % of the loci. Finally, the RH maps were used to anchor 31 BAC contigs (generated by Luo et al. [5]) and 216 sequence scaffold (generated by Jia et al. [21]), more than half of which were not anchored to any previous maps. Clearly, dense AL8/78-RH maps have the potential to anchor and order available BAC contigs [5] and sequence scaffolds [21], which can ultimately lead to an improved and complete genome assembly of the wheat D-genome. This study provides a model for developing dense and accurate molecular maps for completing sequence assembly in large and complex plant genomes where *de novo* sequence assembly is difficult.

## Methods

### Radiation hybrid population for D-genome of *Ae. tauschii*

A radiation hybrid panel consisting of 1,510 lines was developed for the D-genome of *Ae. tauschii* accession AL8/78 (named AL8/78-DGRH_1_) [15]. In brief, seeds of synthetic hexaploid wheat line SW58 (*T. aestivum* L.; 2n = 42; AABBDD; [36], were γ-irradiated to create deletions. The RH_0_ plants obtained from γ-irradiated SW58 seeds were crossed with ‘Langdon’ (LDN; *T. durum* L*.*; 2n = 28; AABB) to develop the RH_1_ (or F_1_) seed (2n = 35; AABBD). The plants generated from these RH_1_ seed constitute the AL8/78-DGRH_1_ panel, where deletions for D-genome markers could be detected due to a hemizygous condition [15]. From this panel of 1,510 RH_1_lines, a subset of 178 most informative lines was selected for this study. Informative lines are defined to harbor at least one deletion, as determined by screening with 35 SSRs covering the D-genome. Five markers from each of the seven D-genome chromosomes were used in the initial screen.

### DArT and SSR genotyping

DNA extraction and SSR genotyping has been described by Kumar et al. [15]. DArT analysis [31] was carried out by Diversity Arrays Technology Pty. Ltd (Yarralumla, Australia; http://www.diversityarrays.com) using 25 μl of DNA (100 ng/ μl) per genotype for a total of 178 lines from the AL8/78-DGRH_1_ panel. An expanded version of the WHEAT 2.6 DArT array was used where coverage for the D-genome of wheat was increased by adding markers from a *Pst*I(*Taq*I) genomic representation of D-genome enriched DNA fractions. The parental genotypes SW58 and LDN were also included in the analysis as positive and negative control respectively. DArT markers present in SW58 and absent in LDN indicate their D-genome specificity and such markers were used to genotype 178 lines of the RH panel. SW58 and LDN were each replicated four times to exclude any markers showing inconsistent results. In total, 641 D-genome specific DArT markers were identified.

### Data analysis and RH map construction

The RH panel was scored for marker presence (1) or absence (0). Only markers showing consistent results for parental genotypes were included in the final analysis. Marker loss or retention frequency was defined as the proportion of RH lines with a marker lost/retained in the AL8/78-DGRH_1_ panel.

The genotyping data comprising of SSRs (which were used to characterize the whole RH panel [15]) and DArTs on 178 RH lines was used to construct RH maps for the whole D-genome using Carthagene 1.2.2 [37]. Initially, all markers were separated into seven groups (each representing a chromosome of the D-genome) using a minimum LOD score of 3. Markers belonging to individual chromosomes were then used for map construction using the following strategy [13, 15, 38, 39]. A set of markers selected based on available genetic and deletion bin information [23, 29, 40, 41] were first anchored. The remaining markers were placed onto the framework map using iterative analysis (for details see, Kumar et al. [13]; Al-Azzam et al. [38]; Seetan et al. [39]). A LOD score of 3 or higher was used to assign markers to a particular RH group or chromosome.

The graphical representation of the RH maps was drawn using MapChart software [42]. To confirm map order accuracy, the AL8/78-RH maps were tested by comparing with:1) a DArT consensus genetic map constructed using more than 100 bi-parental populations [23], 2) a RH map of the D-genome of reference hexaploid wheat’Chinese Spring’ [14], and two SNP-based genetic maps, one with anchored D-genome BAC contigs [5] and another with anchored D-genome sequence scaffolds [21].

As the consensus map by Huang et al. [23] and the RH map by Tiwari et al. [14] have DArT markers with the same nomenclature, these maps were directly compared with the present RH maps, using AutoGRAPH (http://autograph.genouest.org; [43]). However, to identify the common markers in Luo et al. [5], the available DArT sequences were used to BLAST compare against the sequences containing SNP markers mapped by Luo et al. [5]. In the case of Jia et al. [21], the SNP markers were used to anchor the sequence scaffolds onto the genetic map. So to identify the common markers, the DArT sequences were BLASTN compared against the mapped sequence scaffolds.

The available sequences of 530 DArT markers mapped in the present study were provided by Andrzej Kilian of Diversity Arrays Technology Pty. Ltd (http://www.diversityarrays.com). As has been reported in the past that same sequences are represented by different DArT markers [28], so to find out the level of redundancy in those DArT sequences, a Python script was written. The script creates an array, reads the sequences file and maps the sequences to that array, where each sequence is stored in one element in the array. Then a loop is executed over the array to compare each sequence in the array with all other sequences to find the matched sequences.

### Anchoring the RH map to the D-genome BAC contigs and draft sequence

In order to assist in development of a high density marker scaffold with complete coverage from both low and high recombination regions of the D-genome of wheat, as a first step, the RH maps were anchored with a recently developed BAC contig based physical map [5] and draft sequence of the D-genome of *Ae. tauschii* [21]. The sequences of these DArT markers were used to BLAST compare against the sequences of the markers mapped on: 1) AL8/78 × AS75 genetic map, which has been anchored to a BAC contig physical map [5] and 2) sequence scaffolds belonging to a draft sequence of *Ae. tauschii* [21]. The draft genome sequence of *Ae. tauschii* was downloaded from http://gigadb.org. For BLAST analysis, a significant match was declared when there was at least 90 % nucleotide identity for not less than 100 bases and with an e value of not less than *e*^−40^. In case of more than one hits, the most significant hit was considered for analysis.

## Additional file

Additional file 1:
**Table Comparision/anchoring of AL8/78 radiation hybrid (RH) maps with previous RH and genetic maps.** (XLSX 116 kb)

## Abbreviations

BAC: Bacterial artificial chromosome
cR: centi-Rays
DArT: Diversity arrays technology
DGRH: D-genome radiation hybrid
LDN: Langdon
IWGSC: International Wheat Genome Sequencing Consortium
RH: Radiation hybrid
